# Genotype–phenotype correlation in *BRCA1/2* mutation-associated pancreatic cancer

**DOI:** 10.1038/s41416-019-0645-9

**Published:** 2019-12-02

**Authors:** Maeve A. Lowery

**Affiliations:** Trinity St James Cancer Institute, Trinity College Dublin, Dublin, Ireland

**Keywords:** Pancreatic cancer, Cancer genetics, Cancer therapy

## Abstract

Pancreatic cancers occurring in carriers of a pathogenic germline alteration in BRCA1/2 (gBRCA1/2) are assumed to demonstrate homologous recombination repair deficiency (HRD), associated with sensitivity to platinum-based chemotherapy and synthetic lethality with PARP inhibitors (PARPi). However, primary and secondary resistance to these agents occurs even in this highly selected population.

## Main

Pancreatic cancer occurs with increased frequency among carriers of pathogenic germline alterations in BRCA1, BRCA2 (gBRCA1/2) and in additional genes including ATM, PALB2, TP53 and CDKN2A.^1^ National Comprehensive Cancer Network (NCCN) and American Society of Clinical Oncology (ASCO) clinical practice guidelines currently recommend genetic counselling and consideration of germline genetic testing for all patients with a diagnosis of pancreatic ductal adenocarcinoma (PDAC).^2^ The incorporation of universal screening of PDAC patients for pathogenic germline alterations into standard clinical practice provides opportunities for cascade testing and implantation of cancer screening and preventative interventions among unaffected carrier relatives. However, an increasing challenge in the clinic is assessment of the clinical implications of a pathogenic germline alteration (PGA) for an individual patient, both from a prognostic and therapeutic perspective. Despite more widespread availability of rapid and comprehensive genetic testing, our ability to tailor an individual patient’s treatment strategy based on germline genetic findings remains relatively limited. As increasing numbers of PDAC patients elect to pursue germline genetic testing there is a need to ascertain the phenotypic and therapeutic relevance of pathogenic germline alterations in BRCA1/2 and other PDAC-associated genes so as to determine the real-world implications of these results for clinical decision making.

The potential to exploit a PGA for therapeutic benefit relates predominantly to the identification of tumours with a defective DNA damage response (DDR) due to pathogenic germline alterations in genes including PALB2, BRCA1/2 and ATM. This is associated with increased sensitivity to both DNA-damaging agents such as platinum-based chemotherapies and to drugs targeting the DDR pathway including PARP inhibitors (PARPi).^3^ However, the presence of a gBRCA1/2 mutation does not necessarily confer such a phenotype. A recent study of germline and somatic mutational profiling in over 15,000 cancer patients demonstrated biallelic inactivation, zygosity-dependent phenotype and sensitivity to PARP inhibitors only in gBRCA1/2 mutant tumours associated with increased heritable risk in gBRCA carriers.^4^ These data indicate that the therapeutic implications of gBRCA1/2 mutations are lineage-specific and highlight the importance of genotypic–phenotypic correlation when determining therapeutic actionability of pathogenic germline findings.

In this context, the report by Wattenberg et al.^5^ comparing outcomes between 26 gBRCA mutant PDAC patients treated with platinum-based chemotherapy to a matched non-gBRCA mutant control group provides real-world information regarding platinum sensitivity in gBRCA-associated PDAC patients. They report increased overall response rate (ORR) (58 versus 21%) and increased real-world progression-free survival (PFS) (10.1 versus 6.9 months) among gBRCA PDAC patients treated with platinum-based chemotherapy compared with non-gBRCA mutant controls. Notably, gBRCA PDAC patients had substantially greater benefit with first line compared with second line platinum, and no significant difference in ORR or PFS between the gBRCA and control groups was seen when platinum drugs were administered in the second line or greater setting.

Sensitivity to platinum-based chemotherapy in the first line setting is an important determinant of subsequent responsiveness to PARPi in gBRCA-mutant PDAC. The recently reported POLO study evaluated Olaparib as maintenance therapy in patients with metastatic PDAC and gBRCA1/2 mutation; following successful platinum-based therapy patients were randomised to Olaparib or placebo.^6^ Median PFS was significantly longer in the Olaparib-treated arm (7.4 versus 3.8 months) and an improvement in ORR (23.1 versus 11.5%) and median duration of response (24.9 versus 3.7 months) was also seen, although there was no overall survival difference between the arms. This study is the first to demonstrate efficacy of targeted therapy in a genetically selected PDAC population. Previous Phase 2 studies of single-agent PARPi in gBRCA-mutant PDAC as second or subsequent line of therapy have shown limited activity, with responses seen predominantly in patients who had not had progression of disease on prior platinum-based therapy.^7^

Currently available evidence supports the use of platinum-based chemotherapy in the first line setting for patients with gBRCA1/2 PDAC, with consideration of maintenance PARPi following at least 4 months of stable disease or response to treatment. However, as reported by Wattenberg et al.,^5^ over 40% of gBRCA PDAC patients will not respond to platinum-based chemotherapy, and up to 20% will have progression as best response even in the first line setting. This is consistent with findings from the POLO study, where 21.7% of patients progressed on first line treatment and were ineligible for randomisation. Clearly a subgroup of gBRCA PDAC cases do not display the typical homologous recombination repair deficient (HRD) phenotype amenable to therapeutic exploitation. Optimally, this platinum-refractory subgroup could be identified upfront and selected for an alternative treatment approach. Ongoing studies are evaluating the efficacy of first line combination platinum and PARPi treatment in gBRCA PDAC, a strategy which may overcome primary resistance in some refractory patients.^8^ Patients with PDAC and a PGA in BRCA1/2 or associated DDR genes are a rare but important group of patients in whom systemic treatment may be directed at exploiting synthetic lethality with PARPi and potentially with newer agents targeting additional key components of the DDR pathway including ATM, ATR, CHK1/2 and DNA-PK. Successful sequencing and combination of these agents with chemotherapy, radiotherapy and immune checkpoint inhibitors may be facilitated through delineation of the mechanisms underpinning intrinsic and acquired resistance to chemotherapy and PARPi among gBRCA1/2-mutant cancers lacking a HRD phenotype.

## Data Availability

Not applicable.
